# Pandemic influenza A (H1N1) in Saudi Arabia: description of the first one hundred cases

**DOI:** 10.4103/0256-4947.59366

**Published:** 2010

**Authors:** Mohammad A. AlMazroa, Ziad A. Memish, Ali M. AlWadey

**Affiliations:** a From the Field Epidemiology Program, Ministry of Health, Riyadh, Saudi Arabia; b From the Ministry of Health, Riyadh, Saudi Arabia

## Abstract

**BACKGROUND AND OBJECTIVES:**

In April 2009, the World Health Organization (WHO) declared pandemic influenza A (H1N1) “public health emergency of international concern”. On June 11, 2009, WHO raised the pandemic alert level to phase 6, indicating a global pandemic. By December 2009, more than 208 countries and territories had reported swine flu cases. The descriptive epidemiology of the first reported 100 cases of this virus in Saudi Arabia are summarized in this report.

**METHODS:**

Data were collected from 1 June to 3 July, 2009 using a predesigned questionnaire. Questionnaires were filled by Field Epidemiology Training Program residents. Data for the first 100 complete cases of confirmed pandemic influenza A (H1N1) were compiled and analyzed.

**RESULTS:**

The age of reported cases was in the range of 1 to 56 years. The highest percentage of cases was in the age group of 20 to 30 years followed by the age group of 1 to 10 years. Females represented 55% of the cases; imported cases represented 47%, 58% of whom had come via the King Khaled Airport. The most common nationalities most were from Saudi Arabia and the Philippines. The main symptoms were fever (56%), cough (54%), and sore throat and the number of cases was seen to peak from the 27 to 29 June.

**CONCLUSION:**

Pandemic influenza A (H1N1) is still a threat to Saudi Arabia. Thus, comprehensive and effective measures for surveillance and prevention of the disease are needed to control its spread.

Swine influenza during the 1918 flu pandemic infected one-third of the world's population (an estimated 500 million people) and caused approximately 50 million deaths.^1^ In 1976, an outbreak of swine influenza occurred in New Jersey, USA, which involved more than 200 cases, some of them severe, resulting in one death.^2^ In 1988, another fatality was reported as a complication of swine influenza. From 2005 until January 2009, 12 human cases of swine flu were reported in the USA, but none were fatal.^3^ In 2009, cases of influenza-like illness were first reported in Mexico on March 18; the outbreak was subsequently confirmed as pandemic influenza A (H1N1).^4^ Until early May, nearly 600 pandemic influenza A (H1N1) cases had been confirmed in Mexico, including 25 deaths.^5^

In late April, the WHO declared a “public health emergency of international concern” under the rules of the WHO's new International Health Regulations when the first few cases of the pandemic influenza A (H1N1) virus were reported in the United States.^6^,^7^ As of late June, the WHO reported that pandemic influenza A (H1N1) had been confirmed in almost 60 000 people in more than 100 countries, and 263 deaths were confirmed to have been caused by the disease. On June 11, 2009, the WHO raised the pandemic alert level to phase 6 (indicating a global pandemic). By December 2009, more than 208 countries and territories had reported swine flu cases.^8^

Symptoms of the 2009 “swine flu” pandemic influenza A (H1N1) virus in humans are similar to those of seasonal influenza and of influenza-like illness in general. They include fever, cough, sore throat, body aches, headache, chills, and fatigue. However, the 2009 outbreak has shown an increased percentage of patients reporting diarrhea and vomiting.^9^ In children, signs of severe disease include apnea, tachypnea, dyspnea, cyanosis, dehydration, altered mental status, and extreme irritability.^10^ Pandemic influenza A (H1N1) (swine flu) tends to cause high morbidity but low mortality rates (1-4%). The most common cause of death is respiratory failure; other causes of death are pneumonia, high fever leading to neurological problems, dehydration, and electrolyte imbalance. Fatalities are more likely in young children and the elderly.^11^

According to the Saudi Ministry of Health, the number of laboratory-confirmed cases in Saudi Arabia as of December 30, 2009 was 15850, with 124 deaths. This report summarizes the descriptive epidemiology of the first reported 100 cases of this virus in Saudi Arabia.

## METHODS

Data were collected using a predesigned questionnaire. The first 114 confirmed pandemic influenza A (H1N1) cases were identified from the Ministry of Health (MOH) Infectious Diseases Department database during the period ranging from June 1 to July 3, 2009. Questionnaires with cover letters were sent to the designated hospitals for data collection. Patient medical records were the source of data. Questionnaires were filled by Field Epidemiology Training Program (FETP) residents in collaboration with medical colleagues from those hospitals. Data for the first 100 complete cases of confirmed pandemic influenza A (H1N1) were compiled and analyzed using the Epi Info program. None of the first 100 cases were severe cases and there were no deaths.

## RESULTS

**Figure 1:**
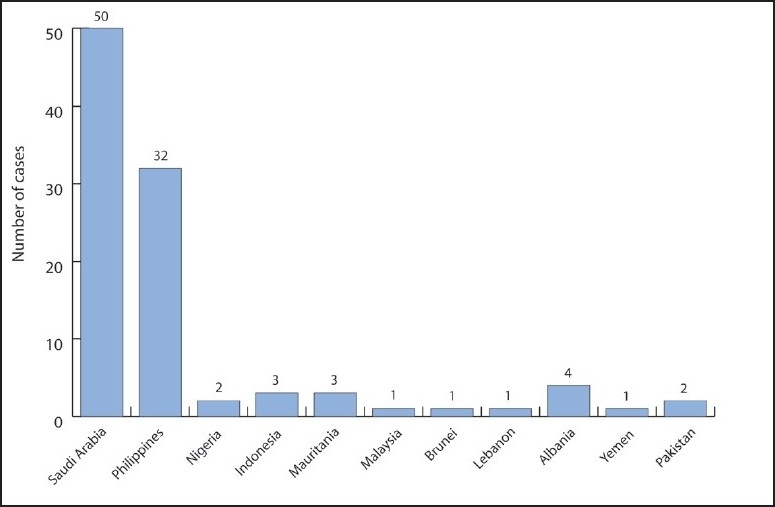
Distribution of cases by national origin.

We were able to complete a questionnaire for the first 100 cases of pandemic influenza A (H1N1) from different hospitals. The age of the cases ranged between 1 and 56 years with a mean (SD) of 24.2 (14.4) years. The highest percentage of cases were in the age group of 20 to 30 years (35%) followed by the age group of 1 to 10 years (22%). There were 45 males and 55 females (Table 1). Fifty-three percent of the cases had some history of contact with diseased persons inside Saudi Arabia while 47% had traveled into Saudi Arabia. The most common national origins were Saudi Arabia and the Philippines (Figure 1).

**Table 1:** Distribution of cases by demographic data.

Variables	Frequency	Percent
Gender		
Male	45	45.0
Female	55	55.0
**Total**	**100**	**100.0**

Age Group (years)		
1-9	22	22.0
10-19	11	11.0
20-29	35	35.0
30-39	19	19.0
40-49	5	5.0
50-59	8	8.0
Mean (SD)	24.2 (14.4)	

**Total**	**100**	**100.0%**

**Figure 2:**
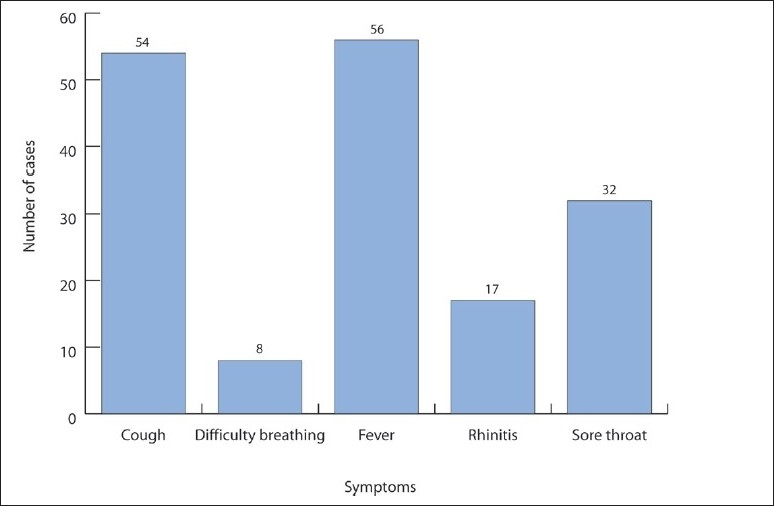
Distribution of cases by reported symptoms.

**Figure 3:**
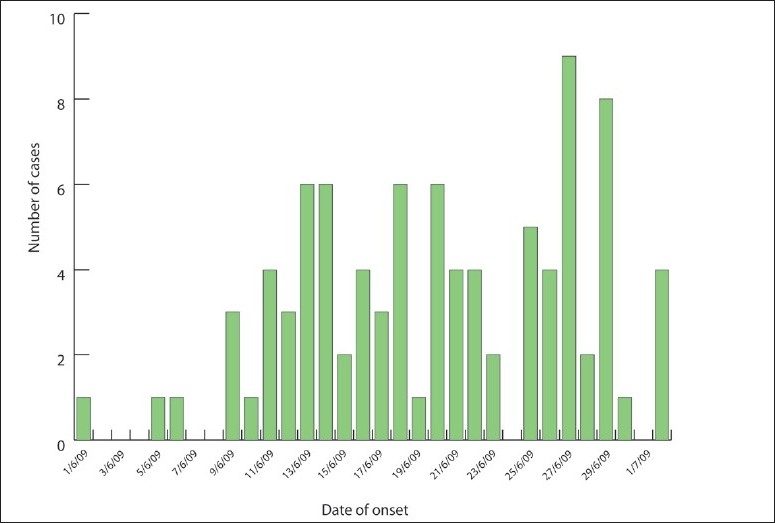
Distribution of cases by date of onset.

**Figure 4:**
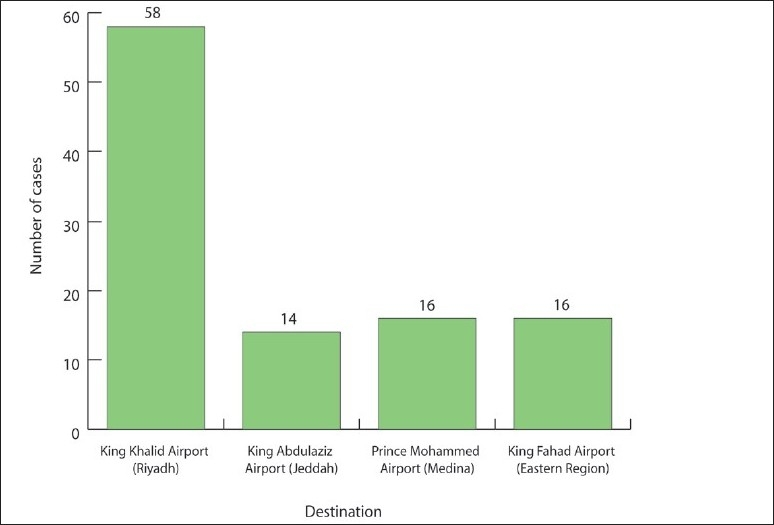
Distribution of cases by arrival destination.

The symptoms reported by the cases were fever (56%), cough (54%), sore throat (32%), rhinitis (17%), and difficulty breathing (8%) (Figure 2). The peak in the number of cases appeared between June 27 and 29. (Figure 3). Fifty-eight percent of the cases had entered the country through King Khalid Airport in Riyadh, 16% through King Fahad Airport in the Eastern region, and 14% through King Abdul Aziz Airport in Jeddah (Figure 4).

## DISCUSSION

As of September 27, 2009, there have been more than 340 000 laboratory-confirmed cases of pandemic influenza A H1N1 2009 worldwide and over 4100 deaths have been reported to the WHO. As many countries have stopped counting individual cases, particularly the milder illnesses, the case count is significantly lower than the actual number of cases that have occurred.^12^ Health authorities in Saudi Arabia kept a high level of alertness in monitoring the situation of pandemic influenza A (H1N1) all over the world especially during winter and around the Hajj season which prevented further spread of the virus locally, regionally, and internationally.

The first 100 cases of pandemic influenza A (H1N1) were mostly among youth aged less than 40 years. A characteristic feature of the pandemic influenza A (H1N1) is that it has mostly involved children and young adults. Similar results were observed in different parts of the world: in Europe, the age of confirmed cases ranged between 1 and 53 years with a median of 23 years.^13^ One of the early studies from the USA showed that although the age of pandemic influenza A (H1N1) patients in the study ranged from 3 months to 81 years, 60% of patients were 18 years of age or younger. In most countries, the majority of Pandemic influenza A (H1N1) cases have been occurring in young people, with the median age estimated to be 12 to 17 years in Canada, the USA, Chile, Japan, and the UK. This age distribution speaks in favor of at least partial immunity to the virus in the older population.^14^

Among the cases in the present study, the main symptoms were fever, cough, and sore throat. H1N1 is a rather mild, self-limiting, upper respiratory tract illness with (or at times without) fever, cough, and sore throat. Up to 50% of all patients present with gastrointestinal symptoms including diarrhea and vomiting. The spectrum of clinical presentation varies from asymptomatic cases to primary viral pneumonia resulting in respiratory failure, acute respiratory distress, multi-organ failure, and death.^15^ The rate of hospitalization could actually be as high as 10% in some cities. Most, but not all, of the hospitalized patients have underlying conditions such as cardiovascular disease, respiratory disease including asthma, autoimmune disorders, obesity, diabetes, or cancer. Pregnant women, especially in their second and third trimester, are also at a high risk for more severe disease.^16^

Nearly one half of all confirmed cases were found to have traveled into Saudi Arabia because of the large number of expatriate workers in different sectors. The country also annually hosts the largest international gathering of the Hajj where 2 to 3 millions gather in a small geographical area. This puts Saudi Arabia in the front line in facing the threat of pandemic influenza A (H1N1). Control measures will not only benefit Saudi Arabia, but will also be of benefit to other countries, especially those with low prevalence, to stop or control the spread of the epidemic.^17^,^18^
